# Complications associated with local anesthesia in dentistry: Review and considerations for a classification proposal

**DOI:** 10.21142/2523-2754-1404-2026-320

**Published:** 2026-10-01

**Authors:** Elmo Sigifredo Palacios-Alva, Jerson J. Palomino-Zorrilla, Lizardo Augusto Saenz-Quiroz, Ivan Calderon-Cortez, Olinda Huapaya-Paricoto, Livia Sonia Zambrano de la Peña, Maria Eugenia Guerrero

**Affiliations:** 1 OMEQ Research Group, Faculty of Dentistry, Universidad Nacional Mayor de San Marcos. Lima, Peru. epalaciosa@unmsm.edu.pe lsaenzq@unmsm.edu.pe icalderonc@unmsm.edu.pe dhuapayap@unmsm.edu.pe szambranod@unmsm.edu.pe mguerreroac@unmsm.edu.pe Universidad Nacional Mayor de San Marcos OMEQ Research Group Faculty of Dentistry Universidad Nacional Mayor de San Marcos Lima Peru epalaciosa@unmsm.edu.pe lsaenzq@unmsm.edu.pe icalderonc@unmsm.edu.pe dhuapayap@unmsm.edu.pe szambranod@unmsm.edu.pe mguerreroac@unmsm.edu.pe; 2 Doctoral Program in Health Sciences, Faculty of Medicine, Universidad Nacional Mayor de San Marcos. Lima, Peru. jerson.palomino@unmsm.edu.pe Universidad Nacional Mayor de San Marcos Doctoral Program in Health Sciences Faculty of Medicine Universidad Nacional Mayor de San Marcos Lima Peru jerson.palomino@unmsm.edu.pe

**Keywords:** local anesthesia, mandibular nerve, dental anesthesia, oral surgery, anestesia local, nervio mandibular, anestesia dental, cirugía bucal

## Abstract

**Background:**

Commonly occurring and known complications associated with local anesthesia and possible treatment modalities have been discussed. The dentist should recognize the symptoms and know that there may be more serious complications. This study aimed to review the literature on accidents and complications associated with dental local anesthetics and propose a classification based on anatomical depth and clinical presentation.

**Materials and Methods:**

A systematic search was conducted in PubMed, Web of Science and Scopus until June 2025. The keywords were based on MeSH and DeCS terms related to dental anesthesia and its complications. In vivo studies, case reports, and case series in oral surgery were included. After reviewing 364 records, 28 studies met the inclusion criteria. Data on study characteristics, type of anesthesia, complications, and treatment were extracted.

**Results:**

Complications were more frequent with inferior alveolar nerve blocks. Local events included puncture fractures, paresthesia, hematoma, ischemia, infections, and trismus. Systemic complications included syncope, toxicity, vasoconstrictive reactions, and allergies. While many resolved spontaneously, others required surgical or pharmacological intervention. A new classification based on the anatomical planes affected (superficial, deep, neurological, and orbitoocular) and systemic categories is proposed, facilitating recognition and treatment.

**Conclusion:**

Local anesthetics, although widely used and generally safe, can cause various complications in oral surgery. The proposed classification systematizes these events and may improve diagnostic clarity, prevention strategies, and clinical decision-making.

## RESUMEN

**Antecedentes:**

Se han analizado las diferentes complicaciones asociadas a la anestesia local y sus posibles tratamientos. El odontólogo debe reconocer los síntomas y tener en cuenta que pueden presentarse complicaciones más graves. Este estudio tuvo como objetivo revisar la literatura sobre accidentes y complicaciones relacionadas con la anestesia local dental y proponer una clasificación basada en la profundidad anatómica y la presentación clínica.

**Materiales y métodos:**

: Se realizó una búsqueda sistemática en PubMed, Web of Science y Scopus hasta junio de 2025. Las palabras clave se basaron en términos MeSH y DeCS relacionados con la anestesia dental y sus complicaciones. Se incluyeron estudios *in vivo*, informes de casos y series de casos en cirugía oral. Tras revisar 364 registros, 28 estudios cumplieron los criterios de inclusión. Se extrajeron datos sobre las características del estudio, el tipo de anestesia, las complicaciones y el tratamiento.

**Resultados:**

Las complicaciones fueron más frecuentes con los bloqueos del nervio alveolar inferior. Los eventos locales incluyeron fracturas por punción, parestesia, hematoma, isquemia, infecciones y trismo. Las complicaciones sistémicas incluyeron síncope, toxicidad, reacciones vasoconstrictoras y alergias. Si bien muchas se resolvieron espontáneamente, otras requirieron intervención quirúrgica o farmacológica. Se propone una nueva clasificación basada en los planos anatómicos afectados (superficial, profundo, neurológico y orbitoocular) y categorías sistémicas, lo que facilita el reconocimiento y el tratamiento.

**Conclusión:**

Los anestésicos locales, aunque de uso extendido y generalmente seguros, pueden causar diversas complicaciones en cirugía oral. La clasificación propuesta sistematiza estos eventos y puede mejorar la claridad diagnóstica, las estrategias de prevención y la toma de decisiones clínicas.

## INTRODUCTION

Local anesthetics have been used in dentistry since 1884, when Hasteld and colleagues first used the technique of blocking the inferior alveolar nerve with cocaine. In 1905, Einhorn synthesized procaine, the prototype of the ester group of local anesthetics. In 1944, lidocaine, the first of the amide group, was invented, significantly reducing the adverse effects associated with these drugs. ^1^

Various accidents and complications related to local anesthesia techniques have also been described, so immediate recognition and diagnosis are essential for timely and effective treatment. ^2^^,^^3^ Therefore, we propose the following classification of local accidents and complications arising from anesthesia techniques in the oral cavity, considering the superficial plane, deep plane, neurological, and orbital planes.

The proposed classification considers the orderly identification of the most superficial levels, such as ischemia necrosis of the oral mucosa at the puncture site, and the deeper levels, such as post-anesthetic injection hematoma. These, in turn, are differentiated from neurological complications, such as paresthesias, which can last for hours, weeks, or months. Finally, there are orbital complications, where cases of temporary blindness have been observed, and in sporadic cases, permanent blindness has occurred.

The objective of this study is to review the literature regarding complications of anesthetics in dentistry and also to present a proposal for the classification of local accidents and complications arising from the application of anesthetics in the oral cavity to systematize and organize in a didactic manner the abundant and dispersed information on this fascinating and vital topic, which is the responsibility of the professional work of all dental surgeons.

## MATERIALS AND METHODS

This study was designed as a systematic exploratory review to map and organize the available clinical evidence on complications associated with dental local anesthesia and to support the development of a new clinical classification.

The methodological approach followed the Population-Concept-Context (PCC) framework, which defined:

- Population (P): Patients undergoing dental procedures requiring local anesthesia
- Concept (C): Local and systemic accidents and complications associated with dental local anesthesia techniques
- Context (C): Clinical settings in dentistry and oral surgery

An electronic search was conducted for the last 12 years, without language restrictions, until October 2025, in the following databases: PubMed, Web of Science, and Scopus. A comprehensive question would be as follows: “What local or systemic complications or adverse events could occur after the administration of dental anesthetics?” In addition, a manual search of the references in articles and gray literature was conducted through Lilacs using the following keywords: (dental anesthesia) AND (complications) AND (oral surgery). For the search strategy, keywords from the Medical Subject Headings (MeSH) and their Spanish and Portuguese equivalents, listed in the Descriptors in Health Sciences (DeCs), were used. The bibliographic search used the following terms (MeSH words): “local anesthesia, mandibular block, Gow Gates, Anesthesia Infiltration, complications, dentistry, oral surgery” (Table 1).

**Table 1:** Search strategy

Database	Search strategy
PubMed	(((((("dental health services"[MeSH Terms] OR ("dental"[All Fields] AND "health"[All Fields] AND "services"[All Fields]) OR "dental health services"[All Fields] OR "dental"[All Fields]) OR ("dentistry"[MeSH Terms] OR "dentistry"[All Fields])) OR Maxillofacial[All Fields]) OR periodont?[All Fields]) OR ("surgery, oral"[MeSH Terms] OR ("surgery"[All Fields] AND "oral"[All Fields]) OR "oral surgery"[All Fields] OR ("oral"[All Fields] AND "surgery"[All Fields]) OR "oral surgical procedures"[MeSH Terms] OR ("oral"[All Fields] AND "surgical"[All Fields] AND "procedures"[All Fields]) OR "oral surgical procedures"[All Fields])) AND complications[Title]) AND ((((((("anesthesia, local"[MeSH Terms] OR (Local[All Fields] AND ("anaesthesia"[All Fields] OR "anesthesia"[MeSH Terms] OR "anesthesia"[All Fields]))) OR ("anesthetics, local"[All Fields] OR "anesthetics, local"[MeSH Terms] OR ("anesthetics"[All Fields] AND "local"[All Fields]) OR "local anesthetics"[All Fields] OR "anesthetics, local"[All Fields])) OR ("anesthesia, local"[MeSH Terms] OR ("anesthesia"[All Fields] AND "local"[All Fields]) OR "local anesthesia"[All Fields] OR ("anesthesia"[All Fields] AND "infiltration"[All Fields]) OR "anesthesia, infiltration"[All Fields])) OR ("infiltration anesthesia"[All Fields] OR "anesthesia, local"[MeSH Terms] OR ("anesthesia"[All Fields] AND "local"[All Fields]) OR "local anesthesia"[All Fields] OR ("infiltration"[All Fields] AND "anesthesia"[All Fields]) OR "infiltration anesthesia"[All Fields])) OR (("mandible"[MeSH Terms] OR "mandible"[All Fields] OR "mandibular"[All Fields]) AND block[All Fields])) OR Gow gates[Author]) OR ("anaesthesia"[All Fields] OR "anesthesia"[MeSH Terms] OR "anesthesia"[All Fields])) AND ("2013/10/27"[PDat] : "2025/10/24"[PDat])
Scopus	( TITLE-ABS-KEY ( "local anesthesia" ) OR TITLE-ABS-KEY ( "local anesthesia" ) OR TITLE-ABS-KEY ( "mandibular block" ) OR TITLE-ABS-KEY ( "gow gates" ) OR TITLE-ABS-KEY ( "Anesthesia Infiltration" ) ) AND TITLE ( complications ) AND ( TITLE-ABS-KEY ( dentistry ) OR TITLE-ABS-KEY ( maxillofacial ) OR TITLE-ABS-KEY ( periodont? ) OR TITLE-ABS-KEY ( "gow gates" ) OR TITLE-ABS-KEY ( "Anesthesia Infiltration" ) OR TITLE-ABS-KEY ( "oral surgery" ) AND PUBYEAR > 2012 AND PUBYEAR < 2024 AND PUBYEAR > 2013 AND PUBYEAR < 2025
Web of Science	"Local Anesthesia" (Topic) or "Local anesthesia" (Topic) or "Anesthetics Local" (Topic) or "Anesthesia Infiltration" (Topic) or "Mandibular block" (Topic) or "Gow gates" (Topic) AND complications (Title) AND dental (Topic) or dentistry (Topic) or Maxillofacial (Topic) or periodont? (Topic) or "Gow gates" (Topic)

Two reviewers (MG, OH) selected studies by independently screening titles and abstracts from the search results, based on the following inclusion and exclusion criteria.

### Inclusion criteria

- Studies related to accidents and complications of local anesthetics in oral surgery.
- Clinical studies conducted in human patients, including case reports and case series.

### Exclusion criteria

- Studies on complications in surgeries on other areas of the body.
- Studies on complications in oncological surgeries.
- Studies on complications in reconstructive and orthognathic surgeries of the maxilla and mandible.
- Animal or phantom studies.
- Survey studies
- Literature reviews, and consensus.

Full texts were then obtained for a final decision based on the inclusion and exclusion criteria. Disagreements were resolved by consensus; if necessary, a third reviewer participated in the discussion until a mutually agreed-upon solution was reached. Studies that met the inclusion criteria and did not meet any of the exclusion criteria were accepted for data extraction.

Three reviewers (SZ, JP, and IC) independently extracted the following data in duplicate: study characteristics (author and year, design, country); sample characteristics (age, sex, number of patients); type of anesthesia; local or systemic complications; and treatment of the complications. Additionally, a data collection matrix was generated (Microsoft Excel for Mac version 16.16.17) for descriptive content.

## RESULTS

### Selection of studies

The search strategy yielded 364 records. After eliminating duplicates, 349 articles were included for review of their titles and abstracts. Of these, 296 were discarded because they were unrelated to the research topic, leaving 53 full-text articles to be evaluated for eligibility. After reading them, the reviewers excluded 29 publications that were literature reviews, editorials, and surveys. Finally, they identified four studies from a reverse search (in the citations of the selected publications), resulting in a total of 28 studies included in the review. The selection process through the different phases of the review is presented in a flowchart (Figure 1), as outlined in the PRISMA 26 declaration, which shows the number of records identified, included, and excluded, along with the reasons for these exclusions.

**Figure 1:**
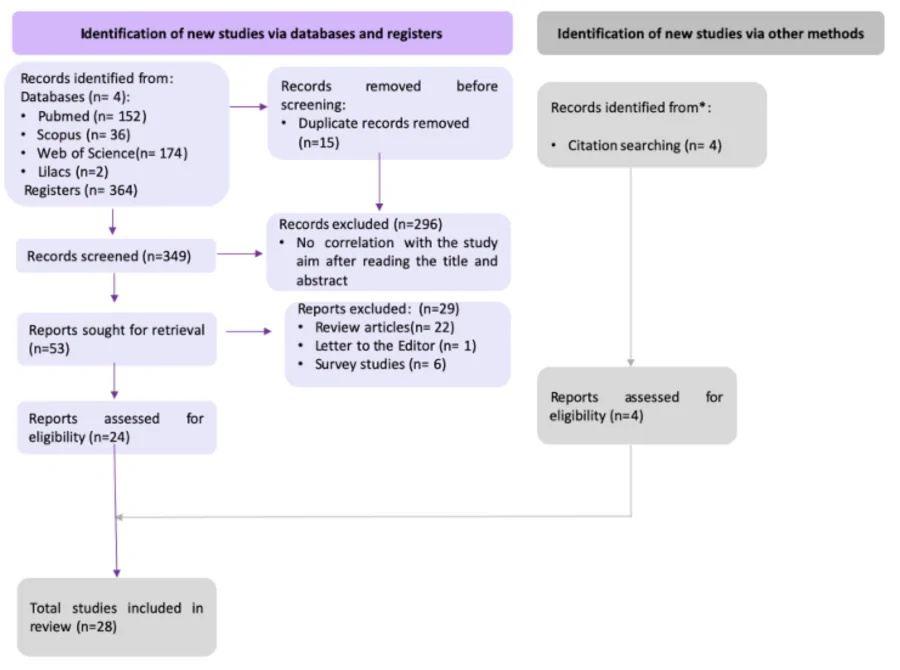
Flow diagram according to the PRISMA statement presenting the study selection process with the number of publications retrieved, included, and excluded.

### Characteristics of the included studies

The characteristics are detailed in Table 2. The review results show that complications and accidents can happen during the use of local anesthetics. From the review, we can conclude that these events are not common. The reports suggest they occur most frequently during inferior dental nerve trunk blocks, followed by procedures in the posterior maxillary areas. The reported incidents may be linked to the application technique, anatomical differences, infiltration into a blood vessel, and the patient's natural response.

**Table 2:** Characteristics of the included studies

	Author / year	Country	Design/No.	Type of anesthesia	Local or systemic complication	Treatment
1	Naghipour et al. ^4^ (2020)	Iran	Randomized control trial/13	Group A: 2% lidocaine (IANB) + 4% articaine for local infiltration. Group B: 2% lidocaine	- Facial edema - Trismus	-Spontaneous remission.
2	Scarano et al. ^5^ (2018)	Italy	Case series/5	Posterior superior alveolar nerve block. Lidocaine 2% with adrenaline 1:100,000	-Vision disturbance and paralysis of the eyelid muscles. - Injection of anesthetic with vasoconstrictor into an artery (palpitations).	-Spontaneous remission.
3	Dilworth et al. ^6^ (2022)	Ireland	Case Report/2	Inferior alveolar nerve block.	-Needle fractures with displacement into the pterygomaxillary space.	CBCT evaluation, and surgical removal in both cases.
4	Tasioudi et al. ^7^ 2022	Greece	Case Report/1	Inferior alveolar nerve block. Articaine 4% with epinephrine 1:100000.	Temporary unilateral diplopia.	Spontaneous remission after 30 minutes.
5	Omori et al. ^8^ 2021	Japan	Case Report/1	Inferior alveolar nerve block.	Needle fracture.	CBCT evaluation, and surgical removal.
6	Galli et al. ^9^ 2021	Argentina	Case Report/1	Truncal nerve anesthesia (Inferior alveolar nerve block)	Needle fracture.	Surgical removal by guided surgery.
7	Gurbanov and Torul. ^10^ 2019	Türkiye	Case Report/1	Infiltrative anesthesia in the hard palate.	Palatal osteonecrosis associated with local anesthesia and actinomycotic infection.	Surgical debridement of affected tissues, antibiotics, and analgesics.
8	Khattab et al. ^11^ 2018	Germany	Case Report/1	Infiltration anesthesia for the upper left canine. Articaine and epinephrine 1:100,000	-Headache. - Diagnosis: cilioretinal artery occlusion with optic disc inflammation.	-Aspirin 100 mg. - Ophthalmoscopic evaluation. -Asymptomatic at 3 months.
9	Biočić et al. ^12^ 2018	Croatia	Case Report/1	Local anesthesia at the maxillary deciduous canine level.	-Immediate swelling of the right cheek. -Acute bacterial infection.	-Systemic corticosteroid. -Clavulanic acid plus amoxicillin for 10 days.
10	Moore et al. ^13^ 2017	USA	Case Report/1	Inferior alveolar nerve block.	-Needle fracture with penetration of the internal carotid artery.	-Removal using an endovascular technique.
11	Gargi et al. ^14^ 2017	India	Case Report/1	Infiltrative anesthesia in the hard palate.	-Painless ulcer in the infiltration area. - Mobile bone fragment in the detached palate, with nasal regurgitation	Soft tissue reconstruction with a full-thickness rotational palatal flap.
12	Kumaresan et al. ^15^ 2017	Malaysia	Case Report/1	Inferior alveolar nerve block. 1.8 ml of 2% lidocaine HCl with epinephrine 1:80,000	-Pallor infraorbital with sharp, stabbing pain. -Paleness of the palate.	Spontaneous remission: infraorbital area at 10 minutes and palate at 20 minutes.
13	Sahin et al. ^16^ 2017	Türkiye	Case Report/1	Inferior alveolar nerve block.	-Needle fracture and displacement into the perivertebral space. -Intense pain and difficulty moving the neck.	CT-guided surgical removal.
14	Odabaşi et al. ^17^ 2017	Türkiye	Case Report/1	Inferior alveolar nerve block. Mepivacaine 3% without epinephrine	Loss of vision in the left eye, diplopia.	Spontaneous remission within 5 minutes.
15	Queiroz et al. ^18^ 2016	Brazil	Case Report/1	Inferior alveolar nerve block.	Needle fracture with cervical migration and proximity to the facial artery.	CT evaluation and surgical removal.
16	Kempster et al. ^19^ 2016	Australia	Case Report/1	Inferior alveolar nerve block. 2% Lidocaine with 1:80,000 adrenaline solution.	Hemifacial spasm and eventual eyelid closure on the contralateral side.	Spontaneous remission after 30 minutes.
17	Lee and Zaid ^20^ 2015	USA	Case Report/1	Inferior alveolar nerve block.	Pin fracture in the right pterygomandibular space.	Surgical removal using a surgical navigation system.
18	Takahashi and Zarazúa (^21^) 2015	Mexico	Case Report/1	Inferior alveolar nerve block.	Pin fracture in the right pterygomandibular space.	CT evaluation with hard and soft tissues, and surgical removal.
19	Malkawi et al. ^22^ 2023	Jordan	Case Report/1	Inferior alveolar nerve block.	Pin fracture in the right pterygomandibular space.	CT evaluation and surgical removal.
20	Moorthy & Stassen ^23^ 2015	Ireland	Case Report/1	Local anaesthetic administered on the buccal and palatal aspects of the tooth involved	Numbness present in the upper left lip from the midline to the commissure, together with numbness of the gingiva in the upper left anterior region of the maxilla.	Seven months reduced sensation. At this stage it was decided to undertake a magnetic resonance imaging. The MRI scan was normal. A course of pregabalin, 100mg three times daily, was prescribed but was unsuccessful.
21	Chisci et al. ^24^ 2013	Italy	Case Report/1	Posterior superior alveolar nerve block. Articaine with epinephrine.	Trochlear nerve palsy: Transient unilateral double vision and diplopia.	Spontaneous remission 2 hours after application of anesthesia.
22	Ezirganli S. and Kazancioglu H. ^25^ 2013	Taiwan	Case Report/2	Inferior alveolar nerve block. Articaine with epinephrine.	Ischemia of the lateral nasal wall, inferior orbital rim, lip, and cheek.	Spontaneous remission occurs between 20 and 30 minutes.
23	Kansu and Yilmaz ^26^ 2013	Turkey	Case Report/1	Local anesthesia and root canal treatment on the left second molar. Articaine with epinephrine.	Balance disorders, tinnitus, a feeling of obstruction, and hearing loss.	Prednisolone, betahistine, trimetazidine, and intratympanic dexamethasone. Remission of symptoms on the 5th day.
24	Pattni ^27^ 2013	United Kingdom	Case Report/1	Inferior alveolar nerve block. 2% lidocaine with 1:80,000 adrenaline	Nocturnal neurological symptoms, tremors, and general numbness.	Progressive spontaneous remission within 24 hours, and lesions healed within a month.
25	Nicot et al. ^28^ 2013	France	Case Report/1	Inferior alveolar nerve block.	Facial cellulitis due to foreign body in the mandibular sub angle.	Drainage by endobuccal approach and cervicotomy. Intravenous and oral amoxicillin plus clavulanic acid.
26	Akifuddin and Khatoon ^29^ 2016	India	Incidence	Only complication events are mentioned, but not all anesthetized patients are mentioned. Systemic complications: syncope (67%), vasoconstrictor complication (15%), hyperventilation (11%), intravascular injection (6%), allergy (2%) Local complications: anesthesia failure (23%), trismus (21%), self-biting (19%), pain at the site (16%), hematoma (6%), transient facial paralysis (5%), others (10%)		
27	Boynes et al. ^30^ 2014	USA	Incidence	Oral infiltrative (41.3%), Inferior alveolar nerve block (30.8%), oral and palatine infiltrative (6.4%), oral infiltrative plus Inferior alveolar nerve block (5.8%), others.	Overall complication rate: 8.1% (14 cases) Self-inflicted soft tissue injuries. Inadequate anesthetic technique. The anesthetic that showed the greatest complications was 2% lidocaine with 1:100,000 epinephrine (8 of the 14 cases).	
28	Barodiya et al. ^31^ 2017	India	Case Report /1	Inferior alveolar nerve block 1.8ml lidocaine with 2% adrenaline	Temporary blindness on the anesthetized side.	Spontaneous remission after 30 minutes.

### Proposed Classification: Conceptual Basis

The classification proposed in this article, beyond isolated clinical manifestations or the type of complication, is based on anatomical depth, tissue involvement, and clinicopathological behavior. ^32^ From a clinical perspective, complications with local anesthesia differ substantially depending on the anatomical plane affected, the structures involved, and the projected clinical course.

Most existing approaches primarily describe dental anesthesia complications in isolation; these events have not been integrated into a unified, anatomically oriented classification. ^32^^−^^34^ Therefore, organizing these events by anatomical depth can provide a logical, reproducible, and clinically meaningful framework that facilitates the diagnosis, management, and prevention of these complications.

### Superficial Complications

These affect the mucosa, submucosa, and superficial soft tissues at or near the injection site. These complications are usually related to vasoconstrictor effects, injection pressure, or local vascular compromise. This category is supported by previous descriptions of ischemic lesions and post-anesthetic necrosis of the oral mucosa and palate. ^10^^,^^32^^,^^33^

### Deep Complications

These affect deep anatomical spaces and are usually associated with procedural factors or anatomical variations. They often require imaging and surgical treatment, as reported in cases of needle fracture and migration into deep spaces. ^6^^,^^10^^,^^17^^,^^35^

### Neurological Complications

This category includes injuries or functional alterations affecting the structures of the peripheral or central nervous system. These complications are clinically characterized by variable onset, unpredictable duration, and the potential for prolonged recovery. Previous studies emphasize the complexity and medicolegal relevance of neurological complications associated with dental anesthesia. ^3^^,^^23^

### Orbito-ocular Complications

These events represent a group characterized by visual disturbances, which may result from vascular diffusion, retrograde arterial flow, or accidental intravascular injection. Although frequently transient, they can have significant clinical and medicolegal implications. ^11^^,^^17^^,^^31^

According to the review, the most common accident involves the fracture of a dental anesthesia needle. Neurological accidents recover quickly without lasting effects. In reviewing published articles, we found that most of the accidents and complications described in the literature have been reported. The contribution of our work is to systematize, organize, and classify local accidents and complications related to the administration of local anesthesia in dentistry (Table 3).

**Table 3:** A proposed classification of local anesthetic accidents and complications in oral surgery.

I. Local anesthetic accidents and complications		
1.1. Superficial Plane	1.2. Deep Plane	1.3. Neurological	1.4. Orbit
Ecchymosis	Hematoma	Paresthesia	Diplopia
Pain during puncture	Needle breakage	Facial paralysis	Exophoresis
Facial skin ischemia	Trismus	Ageusia	Transient strabismus
Self-biting	Emphysema	Dysgeusia	Temporary blindness
Ischemia necrosis	Spread of acute infection		
Frey syndrome	Post-puncture pain		
II. Systemic anesthetic accidents and complications			
2.1. Psychogenic	2.2. Of Toxicity	2.3. Allergic		
Fainting	Relative overdose	Mild: Rush, hives		
Syncope	Absolute overdose	Moderate: Angioneurotic edema	
Hyperventilation syndrome		Severe: Anaphylactic Shock	

## DISCUSSION

The use of local anesthetics is essential and routine in most dental procedures, as they enable comfortable and painless treatment for patients. However, despite their overall efficacy and safety, the administration of these drugs is not without risks and complications; their incidence may be influenced by factors such as age and the anatomical area where the anesthesia is administered.

Ghafoor et al. ^32^ mention complications such as pain, infection, tissue necrosis, nerve damage, transient paralysis of facial nerve, hematoma, and allergy. Additionally, Malamed ^33^ includes tissue peeling and biting of the lips, cheeks, or tongue. Singh et al. ^36^ mention Horner's syndrome (eyelid ptosis, conjunctival vasodilation, pupillary constriction, facial redness, and rashes on the same side where the anesthetic was applied) and temporary blindness. Macouzet ^34^ considers the timing of occurrence: accident (undesirable effects before or during the application of local anesthesia) and complication (effects that occur minutes, hours, days, weeks, or months after the application).

One of the most frequently reported complications is the fracture of the dental needle, which may be retained at or near the pterygomandibular space ^20^^−^^22^ or the perivertebral space ^16^, and which may migrate towards the carotid artery ^13^, towards the facial artery ^18^ or the brain due to constant contractions during phonation, mastication and swallowing at the level of the pharynx and larynx, or due to repetitive contractions of the latter ^35^. The treatment of this complication is by surgical removal of the fractured fragment with the help of cone beam computed tomography ^6^^,^^8^^,^^18^^,^^21^^,^^22^, by guided surgical removal ^9^^,^^16^, or by a surgical navigation system. ^20^

Some complications may experience spontaneous remission, which can vary from minutes to hours, depending on the type of complexity; for example, trismus and edema ^4^, ischemia, infraorbital ischemia ^25^ and palatal ischemia ^15^ may remit within 5 to 30 minutes, while vision loss and diplopia ^17^^,^^24^ may vary between 30 minutes and 2 hours. On the other hand, some complications may experience remission lasting more than 24 hours, such as nocturnal neurological symptoms, tremors, and general numbness. ^27^ However, other injuries such as ischemia necrosis associated with local anesthesia and actinomycotic infection of the palatine fibromucosa may require more extensive procedures, including surgical debridement and antibiotic and analgesic therapy. ^10^

This proposed classification system addresses a significant gap in the literature: there is currently no scientific evidence on the classification of accidents involving local complications of anesthetics in dentistry, with only classifications of systemic complications available. Also, there is currently no standardized system for organizing local complications of dental anesthesia according to anatomical and clinical criteria. Previous approaches have focused primarily on systemic reactions or temporal aspects; however, they do not incorporate anatomical depth or tissue involvement as fundamental organizing principles. Clinically, this proposal enables rapid identification of the affected anatomical plane, directly guiding diagnostic evaluation, treatment strategies, and prognostic assessment, thereby prioritizing therapeutic intervention and improving clinical decision-making. Furthermore, this proposal promotes standardized reporting of these adverse effects, facilitating the development of preventive strategies and therapeutic decision-making, and supporting the development of future clinical guidelines in dental anesthesia. 

Therefore, we propose this new classification as a dynamic model that provides a structured foundation. Additionally, it will promote standardization by enabling adverse events to be consistently identified, labeled, and described. This results in better prevention, as complications can be addressed based on their complexity and relation to the involved anatomical structures. Among its diagnostic advantages, this proposal allows classification by plane and affected anatomical structures, enabling practical, timely management based on injury severity. These are further divided into more specific categories depending on the affected plane (superficial, deep, neurological, or orbito-ocular) and the nature of the systemic event (psychogenic reaction, toxicity, or hypersensitivity). 

To achieve an accurate diagnosis, identifying signs and symptoms that help classify the adverse event is essential, as it improves the clinical record and guides decision-making. For example, diagnosing and managing an ecchymosis of the skin or mucosa differs from handling a hematoma. Ultimately, this will help develop clinical guidelines for diagnosing and treating these complications and create a database to support future research.

## LIMITATIONS

The current review only includes case reports and lacks conceptual tools to systematically categorize these unwanted clinical events. Likewise, after an exhaustive bibliographic search across different databases, it is not guaranteed that all studies have been considered, as the present review only considers those published within the past 10 years, so older research was not included.

## CONCLUSION

Local anesthetics, while essential and generally safe for use in dentistry, can lead to various complications-most frequently reported with inferior alveolar nerve blocks-requiring immediate recognition and timely treatment. Addressing a critical knowledge gap regarding systematic classification, this study proposes a new framework based on affected anatomical planes (superficial, deep, neurological, and orbito-ocular) and systemic categories to systematize the previously dispersed clinical information. This systematic classification is expected to significantly improve diagnostic clarity, promote standardization, and enhance prevention strategies and clinical decision-making for managing adverse events in dental anesthesia.
